# Simultaneous Aurora-A/STK15 overexpression and centrosome amplification induce chromosomal instability in tumour cells with a MIN phenotype

**DOI:** 10.1186/1471-2407-7-212

**Published:** 2007-11-13

**Authors:** Laura Lentini, Angela Amato, Tiziana Schillaci, Aldo Di Leonardo

**Affiliations:** 1 Department of Cellular and Developmental Biology "A. Monroy", University of Palermo, viale delle Scienze, Palermo, Italy; 2 Centro di OncoBiologia Sperimentale, via San Lorenzo 312, Palermo, Italy

## Abstract

**Background:**

Genetic instability is a hallmark of tumours and preneoplastic lesions. The predominant form of genome instability in human cancer is chromosome instability (CIN). CIN is characterized by chromosomal aberrations, gains or losses of whole chromosomes (aneuploidy), and it is often associated with centrosome amplification. Centrosomes control cell division by forming a bipolar mitotic spindle and play an essential role in the maintenance of chromosomal stability.

However, whether centrosome amplification could directly cause aneuploidy is not fully established. Also, alterations in genes required for mitotic progression could be involved in CIN.

A major candidate is represented by Aurora-A/STK15 that associates with centrosomes and is overexpressed in several types of human tumour.

**Methods:**

Centrosome amplification were induced by hydroxyurea treatment and visualized by immunofluorescence microscopy. Aurora-A/STK15 ectopic expression was achieved by retroviral infection and puromycin selection in HCT116 tumour cells. Effects of Aurora-A/STK15 depletion on centrosome status and ploidy were determined by Aurora-A/STK15 transcriptional silencing by RNA interference. Changes in the expression levels of some mitotic genes were determined by Real time RT-PCR.

**Results:**

We investigated whether amplification of centrosomes and overexpression of Aurora-A/STK15 induce CIN using as a model system a colon carcinoma cell line (HCT116). We found that in HCT116 cells, chromosomally stable and near diploid cells harbouring a MIN phenotype, centrosome amplification induced by hydroxyurea treatment is neither maintained nor induces aneuploidy. On the contrary, ectopic overexpression of Aurora-A/STK15 induced supernumerary centrosomes and aneuploidy. Aurora-A/STK15 transcriptional silencing by RNA interference in cells ectopically overexpressing this kinase promptly decreased cell numbers with supernumerary centrosomes and aneuploidy.

**Conclusion:**

Our results show that centrosome amplification alone is not sufficient to induce chromosomal instability in colon cancer cells with a MIN phenotype. Alternatively, centrosome amplification has to be associated with alterations in genes regulating mitosis progression such as Aurora-A/STK15 to trigger CIN.

## Background

Genome instability is a hallmark of the vast majority of human cancers. Nearly 100 years ago, Boveri T.(1914), found that cells containing more than 2 centrosomes segregate their chromosomes abnormally because of the presence of extra spindle poles. Genetic instability is expressed in cancers by an increased rate of a number of different genetic alterations. These different manifestations of genetic instability are classified into two major categories. The first one involves subtle changes in DNA sequences typically represented by microsatellite instability (MIN). The second one is characterized by gains and losses of whole or parts of chromosomes, named chromosomal instability (CIN), and it is considered a driving force for tumourigenesis [1]. MIN occurs in approximately 15% of colon cancers and results from inactivation of the mismatch repair (MMR) system by either MMR gene mutations or hypermethylation of the MLH1 promoter [2]. The mechanisms inducing CIN in cancer and more specifically in colon cancer are only partly understood. At least two possible causes, not mutually exclusive, could be responsible for CIN: mutations in genes encoding mitotic regulators, such as spindle checkpoint proteins, and defects in genes controlling centrosome homeostasis. The presence of mutations of the mitotic checkpoint regulators BUB1 and BUBR1 and amplification of Aurora-A/STK15 (also known as BTAK, Aurora 2, and AIK1) in a subset of human colon cancers have suggested that CIN results primarily from deregulation of DNA replication and mitotic-spindle checkpoints [3]. Aurora-A/STK15 is a member of the Aurora/Ipl1p family of mitotic regulated serine/threonine kinases that are key regulators of chromosome segregation and cytokinesis [4]. The Aurora-A/STK15 gene maps to chromosome 20q13.2, a region commonly found amplified in several epithelial cancers [5]. Overexpression of the Aurora-A/STK15 gene is often associated with centrosome amplification, chromosomal instability, aneuploidy, and transformation. Aurora-A/STK15 overexpression has been detected in a variety of human cancers and cell lines including breast, ovarian, colon, prostate, and neuroblastoma cancer cell lines [6]. Aurora-A/STK15 is also known to play a role in centrosome maturation and migration that are key events to organize a functional mitotic spindle. Centrosomes are crucially involved in the maintenance of genomic stability by organizing a bipolar mitotic spindle ensuring equal segregation of replicated chromosomes to daughter cells [7]. Consequently, defects involving centrosome changes in number, organization, and behaviour, have been found in a variety of solid tumours [8]. By increasing the incidence of multipolar spindles and related spindle abnormalities, these defects can cause chromosome mis-segregation and aneuploidy [9]. The loss of genomic stability appears to be a key molecular and pathogenetic step occurring early in tumourigenesis creating a permissive environment for the occurrence of alterations in tumour suppressor genes and oncogenes [3]. Another protein involved in these processes is the tumour suppressors p53, important regulator of the cell cycle and apoptosis that is frequently inactivated in human cancers. Furthermore, deletion of the p53 gene results in centrosome amplification [10] that is reminiscent of one of the phenotypes induced by elevated expression of Aurora-A/STK15, suggesting a crosstalk between the two proteins. Indeed it has been shown that Aurora-A/STK15 phosphorylates p53 at serine 315 leading to its ubiquitination by Mdm2 and degradation [11]. In co-transfection experiments, p53 suppressed Aurora-A/STK15 induced centrosome amplification and cellular transformation in a transactivation-independent manner. Moreover, it was observed that Aurora-A/STK15 abrogated p53 DNA binding and transactivation activity by phosphorylation of serine 215 [12]. The suppression of Aurora-A/STK15 oncogenic activity by p53 is explained in part by the finding that p53 inhibited Aurora-A/STK15 kinase activity via direct interaction with the latter's Aurora box [13]. Here we show that hydroxyurea induces centrosome amplification in parental HCT116 cells as well as in their isogenic derivatives with both the p53 and the p21^waf1 ^gene disrupted. However, the presence of supernumerary centrosomes alone was not sufficient to induce aneuploidy in these cells. In some cases extra centrosomes, generally originating multipolar spindles, coalesced forming a pseudo bipolar spindle. We observed that after the release from the hydroxyurea block tumour cells lose supernumerary centrosomes. On the contrary, ectopic expression of Aurora-A/STK15 caused supernumerary centrosomes that were maintained over time and likely generated subsequent aneuploidy. These alterations seem to correlate with Aurora-A/STK15 overexpression. In fact Aurora-A/STK15 transcriptional silencing by RNAi decreases both cells with supernumerary centrosomes and aneuploidy in HCT116 cells ectopically expressing Aurora-A/STK15.

## Results

### Hydroxyurea induces extensive but transient centrosome amplification

**Figure 1:**
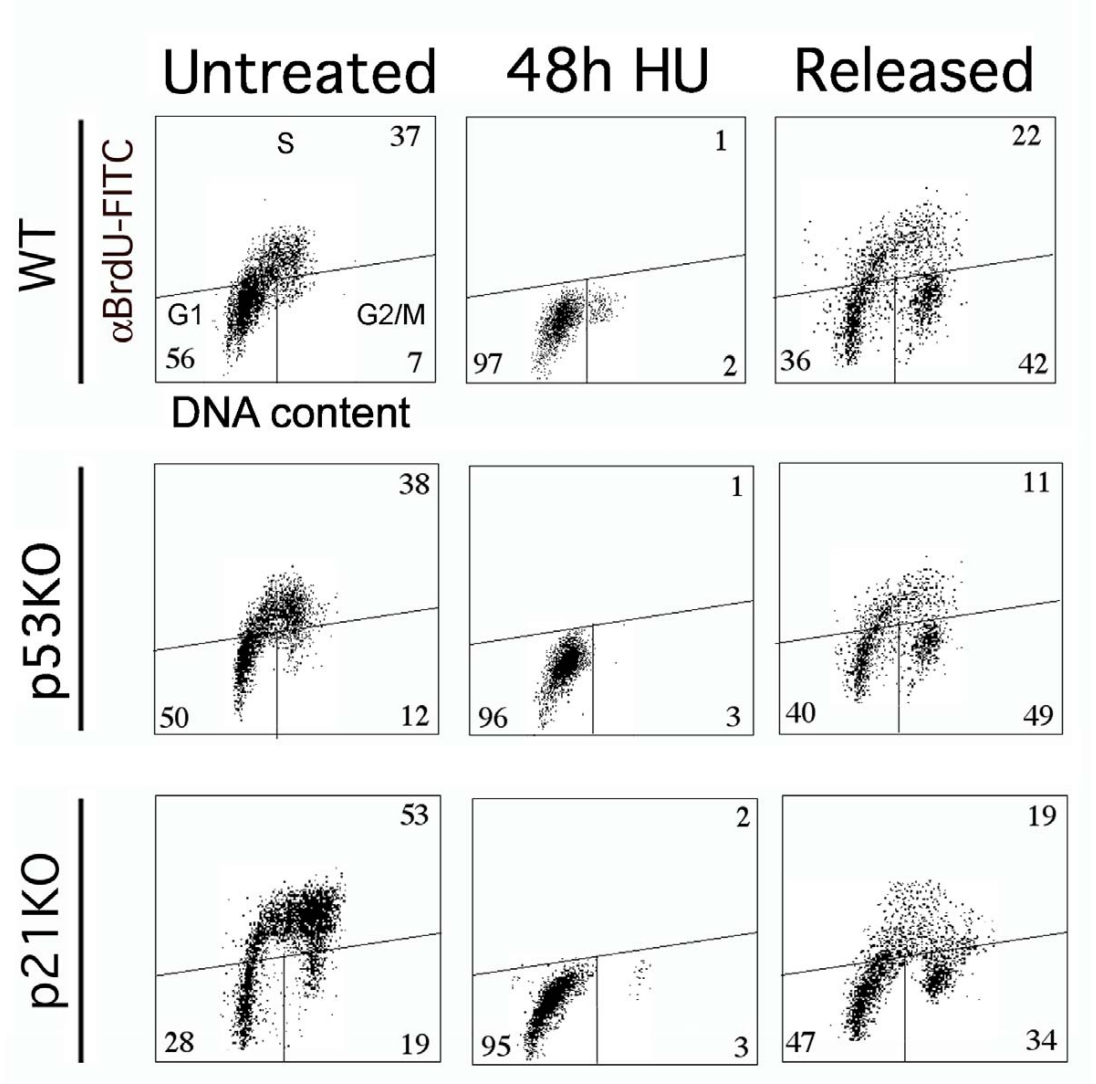
**Cell cycle distribution after HU treatment in HCT116 tumour cells**. Cells were fixed and stained with anti-BrdU-FITC antibody and PI, and analyzed by flow cytometry. In the corners of each panel are reported the percentages of cells in that phase. Representative dot plots of HCT116 tumour cells untreated, treated with HU for 48 hours and released for 24 hours showing a similar profile despite of the genetic background.

**Figure 2:**
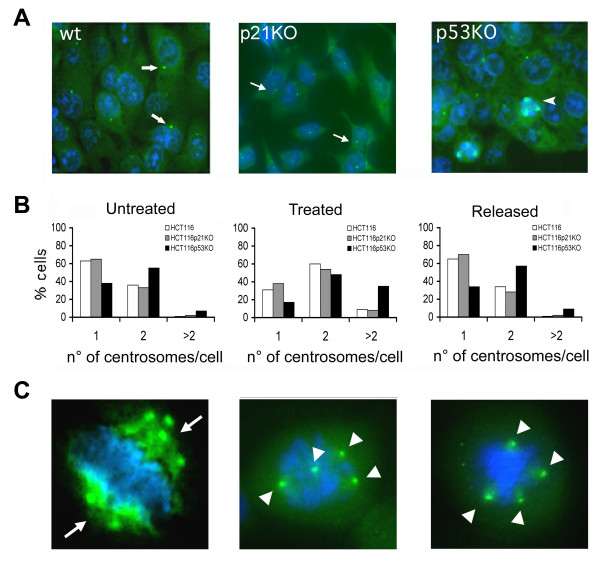
**Centrosome amplification in HCT116 tumour cells after HU treatment**. (A) Centrosome abnormalities revealed by immunofluorescence microscopy detection of γ-tubulin (green) in HCT116 cells treated with HU, nuclei (blue) were stained with DAPI. The majority of HCT -wt and -p21KO cells showed normal centrosomes (arrows). Arrowheads indicate abnormal centrosomes in HCT-p53KO. (B) Histograms indicating percentages of centrosomes numbers in untreated, hydroxyurea treated (48 hours) and released (10 days) cells. (C) Presence of coalesced centrosomes (arrows, likely forming a pseudo-bipolar spindle) and supernumerary centrosomes (arrowheads, likely forming a multipolar spindle) in HCT-p53KO cells.

Unlike the majority of solid tumours the mismatch repair-defective (MIN) colon cancer cells have a normal chromosomal number and do not acquire chromosomal instability (CIN) during the course of many divisions. Indeed MIN and CIN phenotypes are considered mutually exclusive [1]. We have tested if aneuploidy, caused by the presence of extra centrosomes, occurs in tumour cells with a near-diploid karyotype (MIN). As a model system we used the HCT116 chromosomally stable colorectal carcinoma cell line (HCT-wt) and its isogenic derivatives *p53*^-/- ^and p21^-/- ^(HCTp53KO, HCTp21KO), which have both p53 and p21^waf1 ^alleles disrupted [14]. To this aim cells were treated with hydroxyurea (HU), an inhibitor of ribonucleotide reductase activity that accumulated cells at the G1/S border inducing centrosome amplification as previously shown in rodent and human cells [15,16]. Flow citometry analyses showed that cells HU treated for 48 hours arrested in G1/S (figure 1). However, such arrest was transient and when cells were released in drug free medium for 24 hours they entered the cell cycle even though with different kinetics. We observed that a large number of released cells accumulated in the G2/M phase of the cell cycle and the percentage of cells in G2/M was greater for the HCTp53KO cells. Parental HCT116 cells and their derivatives were treated with HU to induce centrosome amplification and any cell with more than two centrosomes was scored as aberrant. Centrosome numbers were estimated in cells growing onto coverslips by detecting the centrosome associated protein γ-tubulin. Immunocytochemistry and fluorescence microscopy revealed that untreated cells did not show more than 1 or 2 centrosomes, with the exception of the HCTp53KO cells which showed 7% of cells with more than 2 centrosomes. HU treatment increased the number of cells with supernumerary centrosomes. Respectively, 8% and 6% of HCT-wt and HCTp21KO cells showed amplified centrosomes. The increase in centrosome numbers (figure 2A–B) has been more evident in HCTp53KO cells (35%), suggesting that lack of p53 allows extensive centrosome amplification. The finding that p21KO cells did not show a large increase in centrosome number as shown by p53KO cells suggests that disruption of the pathway that normally respond to DNA damage, of which p21^waf1 ^is an important component, presumably is not involved in centrosomes amplification caused by HU treatment. In order to establish whether cells with multiple centrosomes could proliferate maintaining these extra centrosomes, we performed immunocytochemistry experiments at various times (one, two and 10 days) after HU treatment. Surprisingly, the number of cells harbouring supernumerary centrosomes decreased as soon as 24 hours of release in drug free medium. By two days after drug removal the numbers of wild type and p21KO HCT116 cells with supernumerary centrosomes were equals to those of untreated cells. On the contrary, at the same time still 20–25% of HCTp53KO showed supernumerary centrosomes. However, after ten days from drug removal also in these cells the percentage of cells with supernumerary centrosomes returned back to the one of untreated cells (figure 2-B). At the end of HU treatment (48 h) we scored the presence of mitotic cells (3%) in p53KO cells. The majority of these mitotic cells (60%) showed coalesced extra centrosomes that presumably will arrange a pseudo bipolar mitotic spindle, while the remaining mitotic cells showed extra centrosomes that likely will arrange multipolar spindles (figure 2-C).

### Ectopic overexpression of Aurora-A/STK15 triggers supernumerary centrosomes and aneuploidy

**Figure 3:**
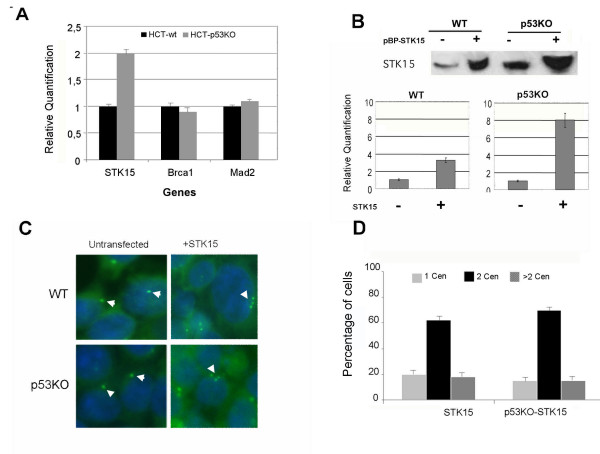
**Gene expression analysis in both HCT-wt and HCT-p53KO cells, presence of supernumerary centrosomes following Aurora-A/STK15 ectopic overexpression**. A) Real time RT-PCR relative quantification shows a two fold expression level of Aurora-A/STK15 gene in untreated HCT-p53KO cells. B) Western blot, upper panel, by anti-STK15 antibody shows increased protein levels of Aurora-A/STK15 in HCT-STK15 and p53KO-STK15 cells. Real time RT-PCR, lower histogram, shows increased expression levels of Aurora-A/STK15 following infection of a retroviral vector encoding the full-length cDNA for Aurora-A/STK15. C) Centrosome analysis by γ-tubulin detection (green), nuclei stained with DAPI (blue). Arrowheads indicate abnormal centrosomes in HCT-STK15 and HCT-p53KO-STK15. D) Graphs summarize the percentage of cells with one, two or more than two centrosomes in untransfected or STK15 overexpressing cells.

**Figure 4:**
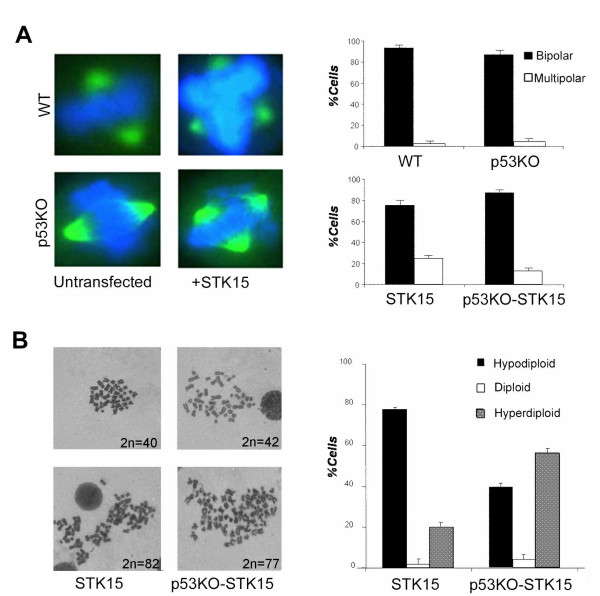
**Alterations of the mitotic spindle and of ploidy following Aurora-A/STK15 overexpression in HCT-wt and HCT-p53KO cells**. A) Presence of multipolar spindles in HCT cells overexpressing Aurora-A/STK15 detected by β-tubulin (green) nuclei are stained with DAPI (blue): graphs summarize the percentages of normal and aberrant spindles in HCT-STK15 and HCT-p53KO-STK15 cells. B) Examples of Giemsa stained aneuploid metaphases both hypodiploid and hyperdiploid; histogram on the right shows percentages of both aneuploid and euploid metaphases in cells overexpressing Aurora-A/STK15.

**Figure 5:**
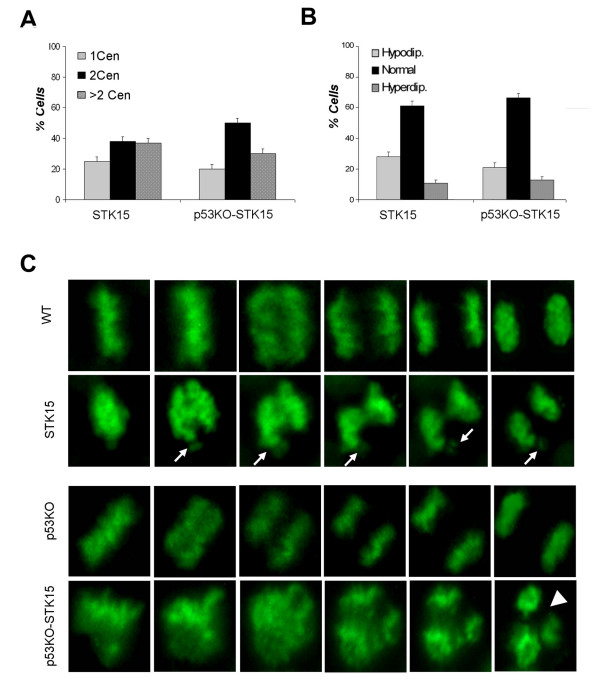
**Centrosome and ploidy alterations after Aurora-A/STK15 transient overexpression and live cell imaging**. A) Graph summarizes percentage of cells with one, two or more than two centrosomes in HCT cells overexpressing STK15 in transient. B) Percentages of aneuploid cells in HCT-STK15 and HCT-p53KO-STK15 C) Images of HCT-wt, HCT-STK15, HCT-p53KO and HCT-p53KO-STK15 during mitosis by time-lapse microscopy. Cells expressed the H2B-GFP gene that decorates chromatin in green. Wild type and p53KO cells exit from mitosis in about 30 minutes. HCT-STK15 cells complete division also in 30 minutes but they show some metaphases with not aligned chromosomes (arrows) that might generate micronuclei. On the contrary HCT-p53KO-STK15 cells completed mitosis after 115 minutes undergoing an asymmetric division (arrowheads).

The results described above suggest that cells in which p53 is not functional and that show a basal level of centrosome amplification could have altered expression of genes necessary for centrosome homeostasis. To investigate this issue further a Real time RT-PCR for Aurora-A/STK15, MAD2 and BRCA1 was performed in both HCT-wt and HCT-p53KO. Real time RT-PCR did not show any changes in expression of MAD2L1 (a spindle checkpoint gene) and BRCA1. On the contrary HCT-p53KO showed a two fold increase of Aurora-A/STK15 expression (figure 3-A). To assess whether Aurora-A/STK15 overexpression is able to induce as well as maintain aneuploidy and extra centrosomes we performed experiments in which this gene was ectopically expressed in HCT-wt and in HCT-p53KO cells. Cells were transfected with the pBPSTR1-Aurora-A/STK15 retroviral vector encoding for the full-length cDNA Aurora-A/STK15 [17] and selected for two weeks in puromycin. Selected cells, named HCT-STK15 and HCT-p53KO-STK15, showed elevated levels of Aurora-A/STK15 protein by western blot (figure 3-B, upper panel) when compared to control cells. As shown by Real time RT-PCR increased protein levels of Aurora-A/STK15 in HCT-p53KO-STK15 cells correlated with higher mRNA expression levels (figure 3-B, lower panel), where transcript quantification is relative to that shown by untransfected cells. Subsequently, we analyzed by immunofluorescence microscopy both HCT-STK15 and HCT-p53KO-STK15 cells to determine if ectopic overexpression of Aurora-A/STK15 was able to induce extra centrosomes (figure 3-C). The presence of supernumerary centrosomes, as revealed by γ-tubulin detection, was observed in 18% and 15% of the cells respectively (figure 3-D). We also observed mitotic spindle alterations in the majority of HCT-STK15 and HCT-p53KO-STK15 mitotic cells as revealed by β-tubulin detection (figure 4-A). These multipolar spindles underlie karyotipic alterations as revealed by conventional cytogenetics performed at two weeks from the infection, that showed the presence of aneuploidy in 97% and 95% of HCT-STK15 and HCT p53KO-STK15 cells respectively (figure 4B, left panel). In particular HCT-STK15 cells showed high percentages of hypodyploid cells (78%), while about 60% of HCT-p53KO-STK15 cells were hyperdyploid (figure 4B). To ascertain whether the effects described above were directly correlated with Aurora-A/STK15 overexpression and not depending on additional genetic changes that occurred during the selection, we over-expressed Aurora-A/STK15 transiently. HCT-wt and HCT-p53KO cells were co-transfected with the pBPSTR1-STK15 vector and the H2B-GFP fusion expression vector [18] to estimate transfection efficiency. After 72 hours from transfection centrosome analysis by γ-tubulin detection revealed an increase of cells with multiple centrosomes (figure 5-A) 37% in HCT-STK15 and 30% in HCT p53KO-STK15 cells. In addition cytogenetics analysis revealed high percentage of aneuploid cells in both HCT-STK15 and HCTp53KO-STK15 where Aurora-A/STK15 is overexpressed, confirming the results obtained in the stably selected cells (figure 5-B).

### Time-lapse video microscopy of cells overexpressing Aurora-A/STK15

To detect the generation of aneuploid cells caused by Aurora-A/STK15 overexpression we analyzed HCT-STK15 and p53KO-STK15 mitoses by time-lapse video microscopy. To visualize mitotic progression we followed H2B-GFP-labeled chromosomes. The H2B-GFP fusion protein decorates chromosomes and allows observation of live cells that fluoresce green under the fluorescence microscope [18]. We measured the duration of mitosis from metaphase to chromosome decondensation in HCT-STK15 and p53KO-STK15 cells compared to their normal counterparts as a control to show that mitosis was not perturbed by the transfection procedure itself. We recorded 14 and 15 mitoses for HCT-STK15 and p53KO-STK15 cells on a total of 220 and 240 cells respectively. Of these mitoses 60% and 85%respectively showed altered mitotic progression. The completion of chromosome alignment on the spindle equator (congression time) was set as T = 0, and the relative times of the onset of chromosome-to-pole movement were determined. Because of possible phototoxicity associated with fluorescence microscopy the time interval between frames capture was limited to one frame every 3 min. HCT-wt and HCT-p53KO cells (figure 5-C) underwent normal metaphase/anaphase transition and completed mitosis in 30 minutes. Also HCT-STK15 cells completed mitosis in 30 minutes, although 8out of 14 of recorded mitosiss they showed some unaligned/lagging chromosomes (arrow) that failed to segregate properly. Such occurrence could originate micronuclei in anaphase and result in the generation of hypodiploid cells. We observed that 13 out of 15 of recorded mitoses in HCT-p53KO-STK15 cells proceeded into anaphase despite the presence of mal-oriented and unaligned chromosomes and did not divide correctly. By anaphase, cells contained errors in chromosome segregation that resulted in a delay (115 minutes) to complete mitosis. Thus, we conclude that Aurora-A/STK15 ectopic overexpression increases the rate of chromosome missegregation and alters the time of the anaphase onset in HCT-p53KO cells.

### Aurora-A/STK15 silencing reduces supernumerary centrosomes, aneuploid cells and cause apoptosis in parental HCT116 cells

**Figure 6:**
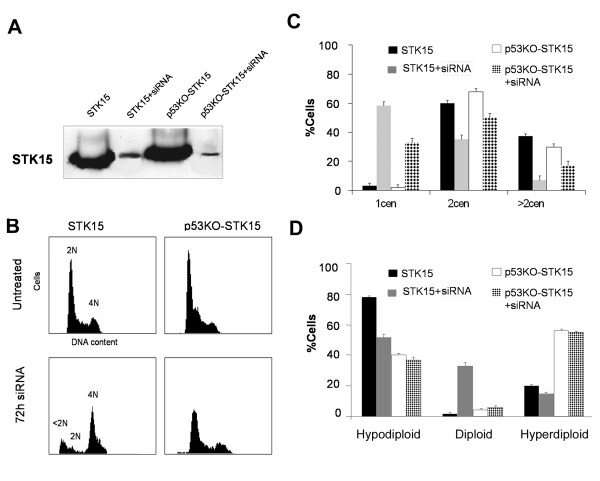
**Effects of silencing Aurora-A/STK15 by RNA interference in HCT-STK15 and HCT-p53KO-STK15 cells**. A) Western blot shows reduced expression of STK15 protein after 72 hours from siRNA transfection. B) FACScan analysis in HCT-STK15 and HCT-p53KO-STK15 cells left untreated and after 72 hours from siRNA transfection. DNA content was revealed by propidium iodide staining. HCT-STK15 cells show the presence of a sub-G1 peak indicating apoptosis in these cells. C) Histogram showing percentages of cells with the indicated centrosome numbers. D) Histogram showing percentages of aneuploid cells in both untransfected and silenced cells. Results in C and D were from three independent experiments (100–200 cells each) and bars indicate standard errors from the mean.

Next we aimed at confirming whether Aurora-A/STK15 overexpression in MIN cells generates chromosomal instability likely via centrosome amplification. To this aim we investigated if depletion of Aurora-A/STK15 expression by RNAi, in cells stably overexpressing Aurora/STK15, was able to reduce supernumerary centrosomes and aneuploidy cells compared to those observed in parental cells. To this aim cells were transfected with specific siRNAs against Aurora/STK15 messenger RNA. Western blot analysis showed that RNA interference caused the reduction of around 90% in the protein level of Aurora-A/STK15 in both HCT-wt and HCTp53KO cells stably overexpressing the kinase (figure 6-A). We also determined the effects of RNA interference on cell cycle distribution. FACScan analyses showed a different effect likely related to the p53 status of the cells. In fact cells with functional p53 arrested mainly in the G2/M phase of the cell cycle, and some of them underwent apoptosis as revealed by the presence of a subG1 peak of DNA content. In contrast we have observed that p53 defective cells seem to enter the cell cycle without appreciable apoptosis (figure 6-B). Then we checked cells for changes in centrosome and chromosome numbers. By γ-tubulin detection we observed in p53 competent cells a reduction of the number of cells both with two and more than two centrosomes as well as an increase of cells with only one centrosome (figure 6-C). Conventional cytogenetics revealed in p53 competent cells a substantial increase of near diploid cells after RNAi (figure 6-D). On the contrary, we did not observe effects of Aurora-A/STK15 transcriptional silencing in p53 deficient cells. Such population showed a slight decrease of cells with more than two centrosomes, but did not show a decrease of the number of cells with chromosome alterations. These last findings confirm a role for Aurora-A/STK15 overexpression in the observed phenotype. Also these results suggest that the differences observed in these two cell types might be caused by the different p53 status and might reflect the fact that once p53^-/- ^cells acquire a new chromosomal asset (aneuploid) these cells do not need Aurora -A/STK15 overexpression to maintain it, so that depletion of this kinase does not affect the number of aneuploid cells

## Discussion

Centrosome amplification and aneuploidy occur in nearly all of solid tumours as well as in leukemia and lymphoma and are considered early features of tumour initiation and progression [19]. However, the presence of multiple centrosome not always results in chromosomal instability [20]. In the attempt to obtain additional evidence on this phenomenon we treated HCT-wt cells harbouring a MIN phenotype and their isogenic derivatives with hydroxyurea that alters the synchrony between DNA and centrosome duplication. Our results show that hydroxyurea induces centrosome amplification in HCT-wt cells and in its derivatives. Such findings are consistent with what previously reported both in rodent and human cells [16,15]. However, in HCT-p21KO cells the frequency of cells with supernumerary centrosomes was much lower than that showed by their p53KO siblings and more similar to that of HCT-wt cells. This last finding suggests that disruption of the pathway normally responding to DNA damage, of which p21^waf1 ^is an important component, likely is not involved in centrosome amplification mediated by hydroxyurea treatment. Hydroxyurea treated cells failed to maintain supernumerary centrosomes and none of the cell lines analyzed became aneuploid despite the initial presence of a large percentage of cells with amplified centrosomes. These results suggest that supernumerary centrosomes can be induced in HCT-wt cells as well as in their derivatives, but they are not maintained over time. Lack of aneuploidy in these cells could be explained by the fact that cells with multiple centrosomes are killed to avoid chromosome instability driven by extra mitotic poles formation. Alternatively, coalesced (clustered) supernumerary centrosomes, may segregate chromosomes correctly likely forming a pseudo bipolar mitotic spindle [8,21]. This could explain why chromosome instability does not occur after release from the HU block, despite of the transient deregulation of the centrosome cycle induced by HU.

These findings suggest that other genes involved in genomic stability and/or centrosome homeostasis should be altered to create a permissive environment that allows HCT116 cells to undergo and perpetuate both centrosome amplification and aneuploidy. By Real-time RT-PCR we looked at changes in expression of some of these genes in HCT-p53KO cells that have a higher basal level of centrosome amplification, and found Aurora-A/STK15 overexpression. Aurora-A/STK15 is localized to centrosomes during interphase and it is essential for their maturation as well as for mitotic progression. It has been also reported that ectopic overexpression of Aurora-A/STK15 in NIH3T3 and immortalized Rat-1 induces cells transformation that generates tumours when implanted in nude mice [17,6]. However, overexpression of Aurora-A/STK15 in primary MEFs does not induce transformation [22] suggesting that additional mutations/alterations are required.

We found that stable ectopic overexpression of Aurora-A/STK15 in HCT-wt and HCT-p53KO cells caused aneuploidy and centrosome alterations that were maintained over time. Transient overexpression of Aurora-A/STK15 confirmed that these effects were directly correlated to the presence of high levels of this kinase in these cells. The same experiments ruled out that such effects depended on additional genetic changes occurring during selection of the cells. These results presented here are also consistent with recent papers showing that Aurora-A/STK15 overexpression causes genetic instability and mammary tumour formation in mice [23,24].

Depletion of Aurora-A/STK15 expression by RNAi in HCT-wt cells stably overexpressing this kinase decreased both aneuploidcells and cells with supernumerary centrosomes confirming a role for Aurora-A/STK15 overexpression in the described phenotype. On the contrary, in HCT-p53KO cells stably overexpressing Aurora-A/STK15 ^-/- ^the reduction of the kinase levels by RNAi did not affect CIN and reduced slightly cells with supernumerary centrosomes. This last result suggests that once aneuploidy has been induced Aurora-A/STK15 overexpression could be no more necessary to sustain proliferation of aneuploid cells.

These differences could be attributable to p53 which can be affected by Aurora-A/STK15 phosphorylation at Ser215 causing in turn abrogation of p53 DNA binding and transactivation activity [12]. In normal cells with wt-p53 functions Aurora-A/STK15 protein levels are controlled at least in part by the Fbxw7 ubiquitin ligase [25]. Loss of p53 could lead to upregulation of Aurora-A/STK15 at protein level mediated by downregulation of Fbxw7 expression. Then, Aurora-A/STK15 ectopic upregulation and inactivation of p53 tumour suppressor pathway could cooperate to drive initial centrosome amplification and aneuploidy in HCT116 cells.

Finally, as recently showed by Sluder and Nordberg in p53^-/- ^MEFs [26] our results call attention to different consequences caused by the presence of multiple centrosomes in relationship to the genetic background of the cell. In particular the results we obtained suggest that multiple centrosome could be a prerequisite to generate aneuploidy in cells that have Auorora-A/STK15 overexpression or that lack of important functions such as those under pRB control [27,15]. On the other hand the outcome of HU treatment in HCT116 cells suggest that to avoid deleterious effects caused by extra poles tumour cells could evolve specific means (i.e.coalescence) to deal with the presence of multiple centrosomes.

## Conclusion

Here we show that the presence of only centrosome amplification is not sufficient to trigger aneuploidy both in HCT116 tumour cells and in their derivatives with the p53 and p21 genes disrupted. The presence in some cases of clustered extra centrosomes, so forming a pseudo bipolar spindle, could in part explain this result. On the contrary, ectopic overexpression of Aurora-A/STK15 causes centrosome amplification that is maintained over time and in turn it results in aneuploidy. Both of these alterations (centrosome amplification and aneuploidy) seem directly correlated with Aurora-A/STK15 ectopic overexpression since its transcriptional silencing by RNA interference decreases both cells with supernumerary centrosomes and reduced the frequency of aneuploid cells. Finally, our findings suggest that centrosome amplification has to be associated to alterations in genes regulating mitosis progression to trigger chromosomal instability.

## Methods

### Cells and cell culture

The human colon cancer cell line HCT116 and its derivatives HCTp21KO and HCTp53KO (a generous gift of Prof B.Vogelstein) which have both p53 and p21 alleles disrupted respectively [14], and HCT-STK15 (expressing Aurora-A/STK15) were cultured in DMEM supplemented with 10% FBS, 100 units/ml penicillin and 0.1 mg/ml streptomycin (Euroclone Ltd UK). HCT116 cells stably expressing Aurora-A/STK15 were obtained by retroviral gene transduction of the pBPSTR1 vector where the Aurora-A/STK15 full length cDNA, (a generous gift of Dr. JR. Bischoff) [17] was cloned. For live cells imaging HCT116 wt and p53KO cells were transfected with the expression vector encoding the H2B histone fused in frame with the Green Fluorescence Protein (H2B-GFP) [18] using the Lipofectamine transfection reagent (Invitrogen). After 24 hours from transfection cells were selected with 1 μg/mL of blasticidin and stable clones were pooled.

### Cell cycle analysis

Asynchronously growing cells were treated with 2 mM hydroxyurea (Sigma) for 48 hours and then released into complete medium without HU. Labelling with BromodeoxyUridine (BrdU) allowed monitoring of cells actively engaged in DNA synthesis. DNA content was determined using Propidium Iodide (PI) staining alone by treating the cells with PBS containing 4 μg/ml of PI and 40 μg/ml RNase. BrdU incorporation (10 μM for 4 hours) was used to determine DNA synthesis of the released cells [28]. Analysis of BrdU labeled cells was conducted as described previously and samples were analyzed on a Beckman Coulter Epics-XL. Experiments were repeated at least twice, 10000 events were analyzed by EXPO32 software for each sample, and representative experiments are shown.

### Determination of ploidy

Asynchronous cells were treated with 0.2 μg/ml colcemid (Demecolcine, Sigma) for 4 hours. Cells were harvested by trypsinization, swollen in 75 mM KCl at 37°C, fixed with 3:1 methanol/acetic acid (v/v), and dropped onto clean, ice-cold glass microscope slides. The slides were air dried and stained with 3% Giemsa in phosphate-buffered saline for 10 min. Chromosome numbers were evaluated using a Zeiss Axioskop microscope under a 100 × objective.

### Immunofluorescence microscopy

To visualize multipolar spindles, cells were grown on rounded glass coverslips and then fixed with 3.7% formaldehyde for 10 min at 37°C, permeabilized with 0.1% Triton X (Sigma) in PBS for 15 min and blocked with 0.1% BSA for 30 min, both at room temperature. Coverslips were incubated with a mouse monoclonal antibody against β-tubulin (Sigma, diluted 1:200 in PBS) overnight at 4°C, followed by a goat anti-mouse IgG-FITC conjugated secondary antibody (Sigma, diluted 1:100 in PBS) for 1 hour at 37°C. For immunostaining of centrosomes of asynchronously grown cells, we used a mouse monoclonal antibody against γ-tubulin (Sigma, diluted 1:4000 in PBS) incubated overnight at 4°C. Cells were washed in PBS buffer and exposed to a FITC-conjugated goat anti-mouse IgG secondary antibody (Sigma, diluted 1:100 in PBS) for 1 hour at 37°C. Nuclei were visualized with 1 μg/ml of 4',6-Diamidino-2-phenylindole (DAPI) and examined on a Zeiss Axioskop microscope equipped for fluorescence, images were captured with a CCD digital camera (AxioCam, Zeiss) and then transferred to Adobe PhotoShop for printing.

### Western blotting

Protein concentration was measured using the Bio-Rad Protein Assay (Bio-Rad Laboratories). Proteins (50 μg) were separated by 10% SDS-PAGE containing 0.1% SDS and transferred to Hybond-C nitrocellulose membranes (Amersham Life Science) by electroblotting. The membranes were sequentially incubated with goat anti-STK15 antibody (Santa Cruz) as primary antibody to visualize Aurora A, and HRP-conjugated mouse anti goat IgG (Santa Cruz) as a secondary antibody. The target protein was detected with enhanced chemiluminescence Western blotting detection reagents (PIERCE). Membranes were stained by Ponceau Red to confirm equivalent loading of total protein in all lanes.

### RNA interference

Twenty-four hours after plating, cells were transfected either with Aurora-A/STK15 siRNA at a final concentration of 60 nM or with control siRNA. A 21-nucleotide duplex siRNA for Aurora-A/STK15 with the following sequence: 5'AUG CCC UGU CUU ACU GUC TT 3' was synthesized by MWG. The day of transfection the siRNA and the transfection reagent (Lipofectamine 2000, Invitrogen) were diluted separately in Opti-MEM (Invitrogen) mixed gently and then incubated for 5 minutes at room temperature. After incubation the siRNA and Lipofectamine 2000 (Invitrogen) were mixed gently, allowed to sit 30 minutes at room temperature to allow complex formation, and added to the plates with 2 ml of D-MEM for 72 hours.

### Live cell time-lapse imaging

Cells were imaged in T25 flasks in CO2-independent medium (GIBCO-BRL) at 37°C. Chromosome segregation was monitored on a heating plate (37°C) with an inverted microscope and a 40x objective on a LEICA inverted microscope equipped with a mercury 100 W lamp. Under UV excitation, cells in metaphase were identified and mitotic progression was monitored every 3 minutes on average. Mitotic progression was documented with a CCD digital camera (AxioCam Zeiss) and then transferred to Adobe PhotoShop.

### Real time RT-PCR

Some genes were selected for Real time RT-PCR experiments. Primers were designed with Primer Express software (Applied Biosystems) choosing amplicons of approximately 70–100 bp. The selected sequences were tested against public databases (NCBI) to confirm the identity of the genes. Total RNA was reverse-transcribed in a final volume of 50 μL using the High Capacity c-DNA Archive kit (Applied Biosystems) for 10 minutes at 25°C and 2 hours at 37°C. For each sample 2 μL of cDNA, corresponding to 100 ng of reverse transcribed RNA, were analyzed by Real time RT-PCR (95°C for 15 sec, 60°C for 60 sec repeated for 40 cycles), in quadruplicate, using the ABI PRISM 7300 instrument (Applied Biosystems). Real time RT-PCR was done in a final volume of 20 μl comprising 1x Master Mix SYBR Green (Applied Biosystems) and 0,3 μM of forward and reverse primers for: MAD2 (Fwd: 5'-GCCGAGTTTTTCTCATTTGG-3'; Rev 5'-CCGATTCTTCCCACTTTTCA-3', Aurka (Fwd:5'-GTTCCC TTCGGTCCGAAAC-3'; Rev 5'-AATCATTTCCGGAGGCTG-3'; BRCA1 (Fwd:5'-CCTTGGCACAGGTGTCCAC-3'; Rev 5' GCCATTGTCCTCTGTCCAGG-3'; GAPDH (Fwd:5'-CTCATGACCACAGTCCATGCC-3'; Rev 5'-GCCAATCCACAGTCTTCTGGGT-3'). Data were analyzed by averaging quadruplicates C_t _(cycle threshold). Levels of RNA expression were determined by using the SDS software version (Applied Biosystems) according to the 2^-ΔΔct ^method. Levels of RNA expression of selected genes were normalized to the internal control GAPDH.

## Competing interests

The author(s) declare that they have no competing interests.

## Authors' contributions

LL carried out RNA preparation for Real time RT-PCR, immunocytochemistry, siRNA experiments and flow cytometry, participated in the design of the study and drafted the manuscript. AA performed HU treatments, Real time RT-PCR in HCT-116 and HCT-p53KO, Flow cytometry, conventional cytogenetics and helped to draft the manuscript. TS did H2B-GFP tansfection, time-lapse videomicroscopy and helped to draft the manuscript, ADL conceived of the study, participated in its design and coordination, and drafted the manuscript. All authors read and approved the final manuscript.

## Pre-publication history

The pre-publication history for this paper can be accessed here:


